# Exposure–Response Analyses of Asbestos and Lung Cancer Subtypes in a Pooled Analysis of Case–Control Studies

**DOI:** 10.1097/EDE.0000000000000604

**Published:** 2017-02-01

**Authors:** Ann C. Olsson, Roel Vermeulen, Joachim Schüz, Hans Kromhout, Beate Pesch, Susan Peters, Thomas Behrens, Lützen Portengen, Dario Mirabelli, Per Gustavsson, Benjamin Kendzia, Josue Almansa, Veronique Luzon, Jelle Vlaanderen, Isabelle Stücker, Florence Guida, Dario Consonni, Neil Caporaso, Maria Teresa Landi, John Field, Irene Brüske, Heinz-Erich Wichmann, Jack Siemiatycki, Marie-Elise Parent, Lorenzo Richiardi, Franco Merletti, Karl-Heinz Jöckel, Wolfgang Ahrens, Hermann Pohlabeln, Nils Plato, Adonina Tardón, David Zaridze, John McLaughlin, Paul Demers, Neonila Szeszenia-Dabrowska, Jolanta Lissowska, Peter Rudnai, Eleonora Fabianova, Rodica Stanescu Dumitru, Vladimir Bencko, Lenka Foretova, Vladimir Janout, Paolo Boffetta, Bas Bueno-de-Mesquita, Francesco Forastiere, Thomas Brüning, Kurt Straif

**Affiliations:** From the a International Agency for Research on Cancer, Lyon, France; b The Institute of Environmental Medicine, Karolinska Institutet, Stockholm, Sweden; c Institute for Risk Assessment Sciences, Utrecht, The Netherlands; d Institute for Prevention and Occupational Medicine of the German Social Accident Insurance – Institute of the Ruhr-Universität Bochum (IPA), Bochum, Germany; e Occupational Respiratory Epidemiology, School of Population Health, University of Western Australia, Perth, Australia; f Cancer Epidemiology Unit, Department of Medical Sciences, University of Turin and CPO Piemonte, Turin, Italy; g INSERM, Centre for research in Epidemiology and Population Health (CESP), U1018, Environmental epidemiology of cancer Team, Villejuif, France; h Université Paris-Sud, UMRS 1018, Villejuif, France; i Epidemiology Unit, Fondazione IRCCS Ca’ Granda—Ospedale Maggiore Policlinico, Milan, Italy; j National Cancer Institute, Bethesda, MD; k Roy Castle Lung Cancer Research Programme, The University of Liverpool Cancer Research Centre, Department of Molecular and Clinical Cancer Medicine, Institute of Translational Medicine, Liverpool, United Kingdom; l Institut für Epidemiologie, Deutsches Forschungszentrum fur Gesundheit und Umwelt, Neuherberg, Germany; m University of Montreal Hospital Research Center (CRCHUM), Montreal, Canada; n INRS-Institut Armand-Frappier, Université du Québec, Laval, Québec, Canada; o Institute for Medical Informatics, Biometry and Epidemiology, University of Duisburg-Essen, Essen, Germany; p Leibniz Institute for Prevention Research and Epidemiology – BIPS, Bremen, Germany; q The Biomedical Research Centre Network for Epidemiology and Public Health (CIBERESP), University of Oviedo, Oviedo, Spain; r Russian Cancer Research Centre, Moscow, Russia; s Public Health Ontario, Toronto, Canada; t Occupational Cancer Research Centre, Cancer Care Ontario, Toronto, Canada; u The Nofer Institute of Occupational Medicine, Lodz, Poland; v The M Sklodowska-Curie Cancer Center and Institute of Oncology, Warsaw, Poland; w National Centre for Public Health, Budapest, Hungary; x Regional Authority of Public Health, Banska Bystrica, Slovakia; y Institute of Public Health, Bucharest, Romania; z Institute of Hygiene and Epidemiology, 1st Faculty of Medicine, Charles University, Prague, Czech Republic; aa Masaryk Memorial Cancer Institute and Medical Faculty of Masaryk University, Department of Cancer Epidemiology & Genetics, Brno, Czech Republic; bb Faculty of Medicine, Palacky University, Olomouc, Czech Republic; cc The Tisch Cancer Institute and Institute for Translational Epidemiology, Icahn School of Medicine at Mount Sinai, New York, NY; dd National Institute for Public Health and the Environment (RIVM), Bilthoven, The Netherlands; and ee Department of Epidemiology, ASL RomaE, Rome, Italy.

## Abstract

**Background:**

Evidence is limited regarding risk and the shape of the exposure–response curve at low asbestos exposure levels. We estimated the exposure–response for occupational asbestos exposure and assessed the joint effect of asbestos exposure and smoking by sex and lung cancer subtype in general population studies.

**Methods:**

We pooled 14 case–control studies conducted in 1985–2010 in Europe and Canada, including 17,705 lung cancer cases and 21,813 controls with detailed information on tobacco habits and lifetime occupations. We developed a quantitative job-exposure-matrix to estimate job-, time period-, and region-specific exposure levels. Fiber-years (ff/ml-years) were calculated for each subject by linking the matrix with individual occupational histories. We fit unconditional logistic regression models to estimate odds ratios (ORs), 95% confidence intervals (CIs), and trends.

**Results:**

The fully adjusted OR for ever-exposure to asbestos was 1.24 (95% CI, 1.18, 1.31) in men and 1.12 (95% CI, 0.95, 1.31) in women. In men, increasing lung cancer risk was observed with increasing exposure in all smoking categories and for all three major lung cancer subtypes. In women, lung cancer risk for all subtypes was increased in current smokers (ORs ~two-fold). The joint effect of asbestos exposure and smoking did not deviate from multiplicativity among men, and was more than additive among women.

**Conclusions:**

Our results in men showed an excess risk of lung cancer and its subtypes at low cumulative exposure levels, with a steeper exposure–response slope in this exposure range than at higher, previously studied levels. (See video abstract at, http://links.lww.com/EDE/B161.)

## Abstract 2

Supplemental Digital Content is available in the text.

Asbestos is a general term for a group of mineral silicate fibers naturally found on all continents; the commercialized types are the serpentine mineral chrysotile (white asbestos) and the amphibole minerals amosite (brown asbestos), anthophyllite, crocidolite (blue asbestos), and tremolite.^1^ Asbestos fibers are generally considered strong, flexible, stable, heat-resistant, and durable; they have therefore been attractive for a wide range of industrial applications for over a century. Consequently, large groups of workers have been (and still are, in many countries) exposed to asbestos, for example in the insulation, textile, cement, roofing, and refractory industries. The highest exposure levels have been measured among workers manufacturing asbestos products or employed in asbestos mining and milling operations.^2^ Asbestos has been banned successively since 1980s in many countries due to its adverse health effects.^3^ Nevertheless, exposure may still occur when buildings insulated with asbestos are demolished, when asbestos is removed from any type of structure, and during maintenance and repair of asbestos-containing materials.^2^ The World Health Organization (WHO) estimated in 2006 that 125 million workers worldwide are still exposed to asbestos.^4^

The International Agency for Research on Cancer (IARC) Monographs Programme evaluated the carcinogenicity of asbestos in 1973, 1977, 1987, and 2011; the Working Group concluded in the most recent evaluation (Vol. 100C) that all forms of asbestos cause mesothelioma and cancer of the lung, larynx, and ovary,^3^ and made no distinction by lung cancer cell type when evaluating asbestos carcinogenicity to the lung.

Lung cancer is the most common cancer globally.^5^ Tobacco smoking is well established as the main cause; for instance, in the United Kingdom, an estimated 85% of lung cancers in men and 47% of lung cancers in women are attributable to tobacco smoking.^6^ Asbestos is the most important occupational carcinogen, and lung cancer is the most common asbestos-related cancer.^7^

Asbestos was the first occupational exposure to be suggested to have a joint effect with smoking.^8^ Several studies and reviews have supported this hypothesis, but the type of interaction (additive or multiplicative) has been debated.^9–13^

Here, we used a pooled dataset of lung cancer case–control studies conducted in Europe and Canada (the SYNERGY project) to estimate lung cancer risk related to occupational asbestos exposure, and its interaction with smoking. The objectives of this work were to (1) estimate the lung cancer risk associated with quantitative indices of occupational asbestos exposure by sex, while adjusting for smoking; (2) assess the exposure–response relationship for asbestos and lung cancer by sex, major subtype, and smoking status; and (3) assess the joint effect of asbestos exposure and smoking on an additive and multiplicative scale.

## METHODS

### The SYNERGY Project

Fourteen case–control studies on lung cancer from Europe and Canada (see eTable 1 at http://links.lww.com/EDE/B144) were pooled in the SYNERGY project to study joint effects of occupational carcinogens, including asbestos, and smoking in relation to lung cancer risk. The studies LUCA and LUCAS were restricted to men, and PARIS to regular smokers with squamous-cell lung carcinoma (SQLC) and small-cell lung carcinoma (SCLC). Participation rates were 62%–98% (mean, 83%) among cases and 41%–100% (mean, 70%) among controls. All studies collected lifetime smoking histories and complete occupational histories, except MORGEN. MORGEN is a case–control study nested in the European Prospective Investigation into Cancer and Nutrition (EPIC) study in the Netherlands, where 45% of those invited completed a questionnaire at recruitment.

The data were collected in 1985–2010, and almost all interviews with study participants were conducted face-to-face. LUCAS and MORGEN collected data using self-administered questionnaires, and women in MONTREAL and some participants in TORONTO were interviewed by phone. Next-of-kin were interviewed for most cases and some controls in LUCAS and some participants in ICARE and MONTREAL (9% of cases, 6% of controls). Controls were individually or frequency-matched to cases by sex and age, and mainly recruited from the general population (79%). Lung cancer subtypes were classified according to WHO guidelines after histological or cytological confirmation. Reference pathology was performed for the German cases.^14^ Ethical approvals for the original studies were obtained in accordance with legislation in each country, and in addition from the IARC Ethics Committee. More information about the SYNERGY project is available at: http://synergy.iarc.fr.

Occupational data consisted of a list of employment periods for every study subject. For every period, job and industrial activity had been recorded, coded respectively to the International Standard Classification of Occupations from 1968 (ISCO-68) and the International Standard Classification of Industries, Revision 2, along with the start and end years.

### Assessment of Occupational Asbestos Exposure

Quantitative measurements of fibers (71,816) from 14 countries (mainly Germany, the UK, Canada, Italy, France, and Norway) were entered into the project-specific exposure database ExpoSYN according to a standardized protocol.^15^ Most data points were determined by phase-contrast microscopy (>95%). It can be assumed that most data represented chrysotile (67%). Regarding measurement strategies, 53% of the measurements were considered “representative,” 9% “worst case,” and 38% “unknown.”^15^ All measurements were linked to a standardized (ISCO-68) job title. Statistical models were applied to the personal measurements (27,958) collected in 1971–2009 to develop a project-specific quantitative job-exposure-matrix (SYN-JEM) for occupational asbestos exposure. Some measurements were attributed to jobs clearly unrelated to asbestos exposure, like teachers; we assumed these to represent exceptional situations, which should not be generalized to all individuals in that job. Therefore, a semiquantitative general population job-exposure matrix based on ISCO-68 codes (DOM-JEM) was used in the model, where every job was rated as nonexposed (=0), low exposed with regard to exposure intensity or high exposed with low exposure probability (=1), or high exposed with high-exposure probability (=2). Jobs considered to be nonexposed in DOM-JEM were set to 0 fibers per milliliter (ff/ml) in SYN-JEM, disregarding actual measurements, if any. When there were <5 measurements for a specific job, the geometric mean estimate of all jobs within the same unit or major group was applied, so the job estimate was based on information from the most similar jobs. Because every job was expert-rated as being non-, low-, or high exposed, an exposure level for every potential job could be calculated, even in the absence of measurements for that particular job. Additional SYN-JEM model specifications and sensitivity analyses using alternative models are described elsewhere.^16–18^ In brief, for all countries and occupations together, we implemented a linear historical trend with an annual decrease of fiber concentrations of −10.7% before ban implementation and no further downward trend after ban implementation, and an exposure ceiling before 1975 to avoid unrealistically high estimates due to unrestrained back-extrapolation to periods when actual measurements were not carried out. Linking the occupational histories of the participants to SYN-JEM generated individual job-, region-, and year-specific estimates of the average intensity of asbestos exposure during a standard 8-hour working day in ff/ml. Cumulative asbestos exposure (expressed as ff/ml-years) was defined as the average exposure intensity in a particular job multiplied by the years of employment, and totaled over the working life of the participants.

### Statistical Analyses

Unconditional logistic regression models were fitted to generate odds ratios (ORs) and 95% confidence intervals (CIs) of lung cancer associated with various indices of asbestos exposure. The subjects classified as nonexposed were the reference category in each of the analyses.

Three strategies for adjustment were applied: the first model (OR1) adjusted for age group (<45, 45–49, 50–54, 55–59, 60–64, 65–69, 70–74, 75+ years) and study; the second model (OR2) also adjusted for tobacco smoking as a continuous variable (log[cigarette pack-years + 1]) and for time-since-quitting smoking cigarettes (current smokers; quitting 2–7, 8–15, 16–25, 26+ years before diagnosis/interview; never-smokers); and the third model (OR3) also adjusted for ever-employment in a “list A” job (yes/no). “List A” is a list of occupations and industries known to present an excess risk of lung cancer, compiled by Ahrens and Merletti^19^ and updated by Mirabelli et al.^20^ Here, we modified “list A” so that jobs originally included solely because of asbestos exposure were excluded, to avoid potential over-adjustment. Examples are asbestos cement product makers, insulators, some jobs in mining and quarrying, and some jobs in manufacture of nonmetallic mineral products not elsewhere classified (e.g., other crushers, grinders, and mixers; beam warpers; loom threaders; fabric examiners and repairers; spinners and winders).

Current smokers were people who had smoked >1 cigarette per day for >1 year, including those who had stopped smoking in the 2 years before diagnosis/interview. Cigarette pack-years were calculated as: ∑duration × average intensity per day/20.

We used kernel plots to describe the distribution of cumulative asbestos exposure among cases and controls. Cumulative asbestos exposure in ff/ml-years was categorized according to quartiles of its distribution in controls for the main exposure–response analyses. In the analyses stratified by lung cancer subtype and smoking status, we used two exposure categories (below and above the median) because the number of observations was limited.

*P* values for linear trend were obtained by applying a logistic regression model including the respective continuous variable. The trend was calculated among all subjects and among exposed subjects only.

We examined robustness of results by sensitivity analyses as follows:

1. excluding one study at a time, to see if any specific study largely influenced the overall result;
2. excluding one industry at a time, to see if any specific industry largely influenced the overall result;
3. stratification by hospital- and population-based studies, to assess if associations differed by study design;
4. restricting the study base to blue-collar workers, to limit potential residual confounding from socioeconomic factors;
5. restricting the analyses to workers who started working in 1960 or later, as exposure data were scarce before the 1960s and exposure estimates in SYN-JEM may be affected by larger uncertainty;
6. excluding “laborers not elsewhere classified” (ISCO 9–99.10) because they represent a substantial proportion of exposed workers in some of the studies, to see if their inclusion had unduly influenced the results.

A multinomial logistic regression model and a likelihood ratio test were used to explore heterogeneity between the three major lung cancer subtypes in relation to a categorical variable of cumulative asbestos exposure.

Lagging of cumulative exposure was applied, in which exposure in the 5, 10, 15, or 20 years before diagnosis/interview was disregarded. As results did not differ by lag-times, we used unlagged models in the main analyses.

The slope of the exposure–response relationship reflects the average excess relative risk per fiber-year. It was obtained from a linear OR model adjusted for study, “list A” occupations, time-since-quitting smoking cigarettes, and smoking pack-year categories using maximum likelihood estimation and was expressed as *K*_*L*_ * 100, that is, 100 times the excess relative risk per fiber-year.

We performed additional spline analyses using nonparametric smoothing as implemented in the R package *mgcv* to assess in more detail the shape of the exposure–response relationship. The optimal smoothing parameter was selected based on generalized cross-validation and under the assumption that the total degrees of freedom required for a biologically plausible model would not exceed 3. 95% CIs for ORs were derived by simulation from the posterior distribution of the model coefficients, performing random draws from a multivariate normal distribution parameterized by the estimated mean vector and estimated covariance matrix of the model coefficients.

Meta-analyses were conducted to explore study-specific ORs using the Stata command “metan,” where the extent of heterogeneity between OR estimates was assessed as a percentage (*I*^2^).^21^

We assessed the additive interaction between smoking and asbestos by estimating the relative excess risk due to interaction.^22^ We again used a linear OR model adjusted for covariates, and bootstrapped CIs for the excess risk due to interaction. Departure from multiplicative interaction between smoking and asbestos was assessed by testing the asbestos–smoking interaction term in the logistic model.

We conducted statistical analyses using SAS, version 9.3 (SAS Institute, Cary, NC); STATA, version 12.1 (StataCorp, College Station, TX); and R, version 3.2.

## RESULTS

We omitted study participants with incomplete data on covariates (804 cases, 848 controls), leaving 16,901 lung cancer cases (4,752 lung adenocarcinoma, 6,503 squamous cell carcinoma, 2,730 small-cell carcinoma, 2,822 other/unspecified lung cancer cell types, 94 not available) and 20,965 controls for the analyses.

### Characteristics of Subjects by Exposure Status

Table 1 shows characteristics of study participants by asbestos exposure status, disease status, and sex. Smoking status differed by asbestos exposure status; nonexposed were more often never-smokers among both men and women. Lung cancer pathology also differed by asbestos exposure status; adenocarcinoma was less frequent and squamous-cell carcinoma more frequent among asbestos-exposed compared with nonexposed men and women. More cases with asbestos exposure ever worked in other occupations with an anticipated lung cancer risk (20% in men) than controls (15% in men) or nonexposed cases (5% in men).

**TABLE 1.**
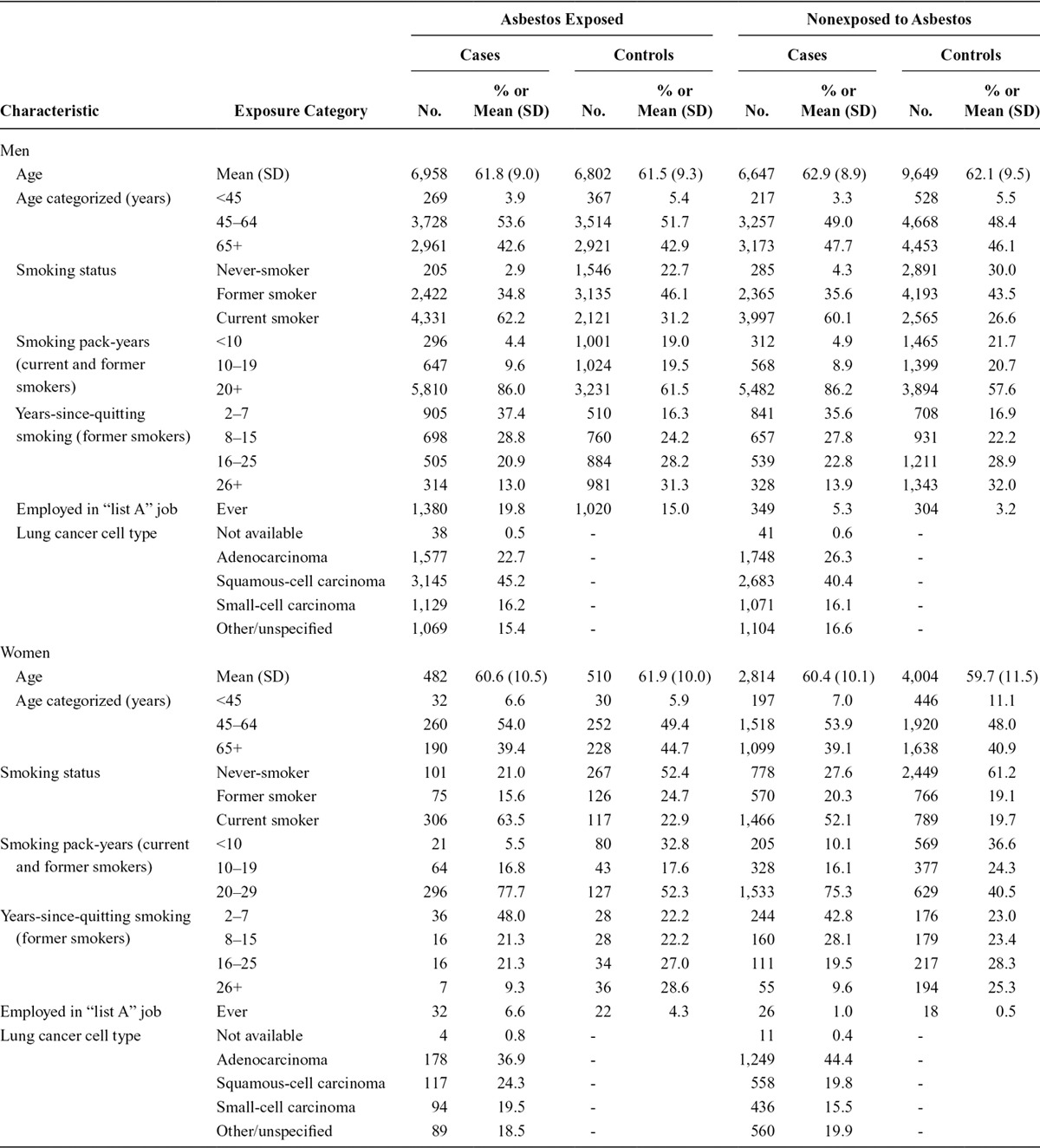
Descriptive Characteristics of the Study Participants (16,901 Lung Cancer Cases, 20,965 Control Subjects) by Asbestos Exposure Status

### Asbestos Exposure

At some time, 44% of cases (51% in men, 15% in women) and 35% of controls (41% in men, 11% in women) had been exposed to asbestos at their workplace. The exposure prevalence among male blue-collar workers was 63% in cases and 58% in controls. eTable 1 (http://links.lww.com/EDE/B144) displays the period for which asbestos exposure was assigned to workers in different studies; it does not reflect when asbestos was banned as some jobs continued to be exposed after the ban. Prevalence of asbestos exposure among control subjects by study and sex, omitting “laborers not elsewhere classified” and restricting to DOM-JEM high levels of asbestos exposure, is displayed in the eTables 2 and 3 (http://links.lww.com/EDE/B144).

**FIGURE 1.**
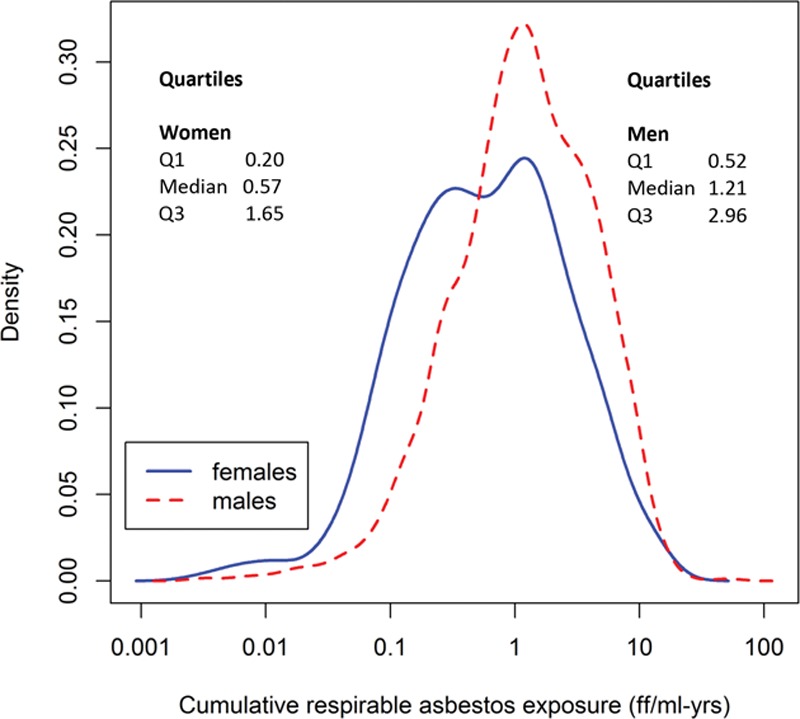
Kernel plot showing distributions of cumulative asbestos exposure in exposed control subjects among women and men in the SYNERGY project.

Figure 1 shows the distribution of fiber-years in exposed control subjects by sex. Overall, women were exposed to lower cumulative levels (median, 0.57 ff/ml-years) than men (median, 1.21 ff/ml-years).

### Lung Cancer Risk Associated with Asbestos Exposure

The ORs for lung cancer associated with ever occupational asbestos exposure changed after adjustment for smoking and for other occupational exposures. In men, OR1, from the model adjusted for age group and study, was 1.43 (95% CI, 1.37, 1.50); OR2, also adjusted for smoking, was 1.29 (95% CI, 1.22, 1.36); and OR3, also adjusted for ever-employment in a “list A” job, was 1.24 (95% CI, 1.18, 1.31). Among women, OR1 was 1.37 (95% CI, 1.19, 1.58), OR2 was 1.13 (95% CI, 0.97, 1.33), and OR3 was 1.12 (95% CI, 0.95, 1.31). In Table 2 and all subsequent analyses, we present OR3 unless otherwise stated.

**TABLE 2.**
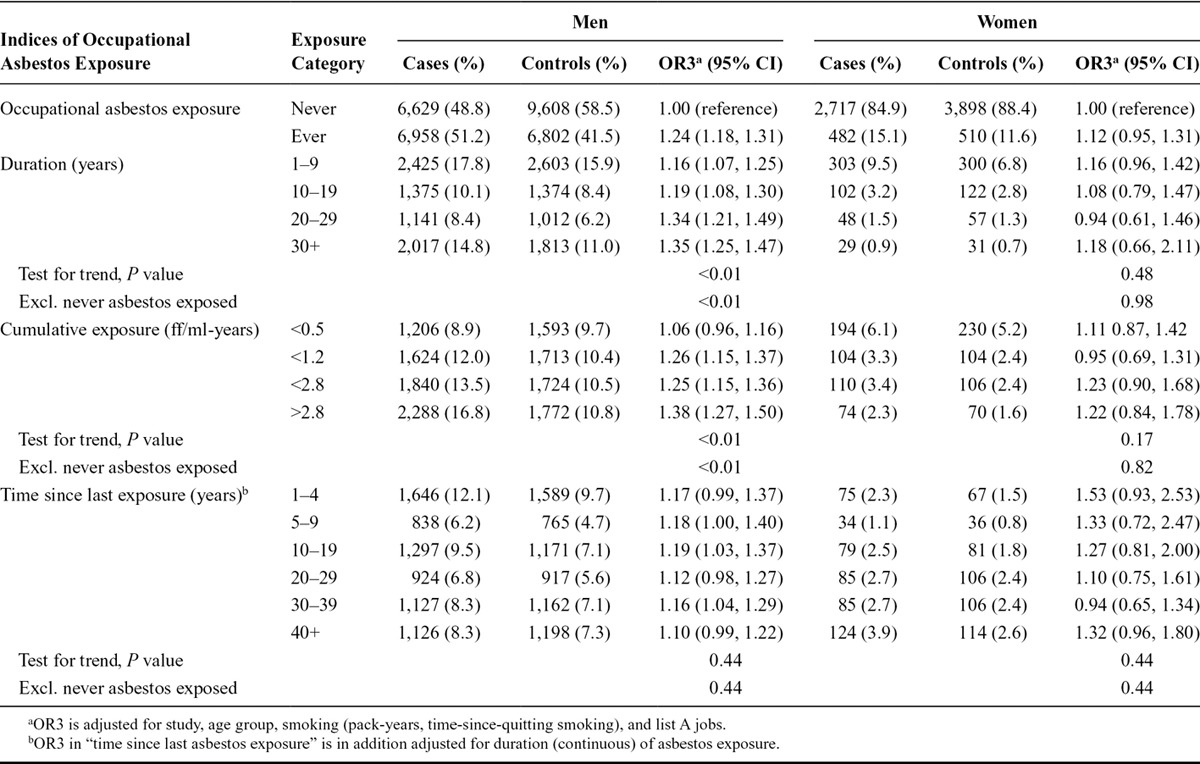
Lung Cancer ORs and 95% CIs in Relation to Indices of Occupational Asbestos Exposure in the SYNERGY Study, 1985–2010

In men, ORs across most exposure categories by duration and cumulative exposure were increased compared with the reference category of never-exposed to asbestos (Table 2). Only the first quartile of cumulative dose (<0.5 ff/ml-years) showed no increased risk (OR, 1.06; 95% CI, 0.96, 1.16). “Time since last exposure” was also adjusted for duration and showed ORs between 1.10 and 1.19, with no time trend (*P* = 0.44).

In women, based on smaller numbers of exposed subjects and a lower median exposure level, no increased ORs were observed (Table 2).

**FIGURE 2.**
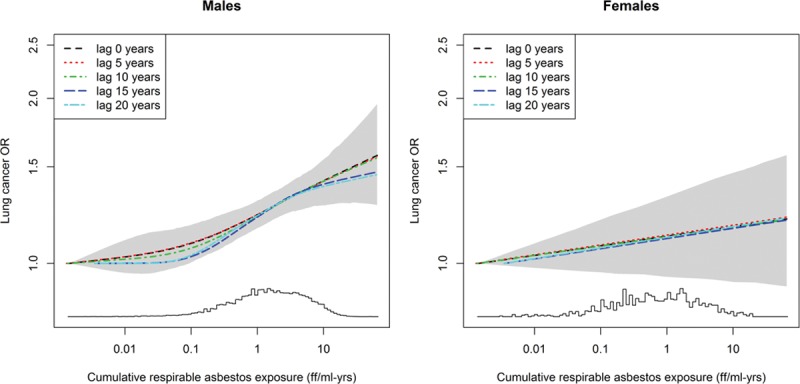
Exposure–response relationship among men and women for cumulative asbestos exposure with different lag periods applied and 95% CIs around the 0-year-lag curve, adjusted for study, age group, cigarette pack-years, time-since-quitting smoking, and ever-employment in a “list A” job. The histograms on the *x* axis show how the study populations are distributed.

Nonparametric exposure–response analyses showed marginal support for a nonlinear exposure–response association among men, which was larger for models with longer lag-times, while the exposure–response was linear among women (Figure 2).

Study-specific results showed no heterogeneity between studies, with *I*^2^ = 0% in both men and women (eFigures 4 and 5; http://links.lww.com/EDE/B144, which show forest plots and heterogeneity tests).

The exposure–response slope (*K*_*L*_ * 100) among all men was 6.1 (95% CI, 4.1, 8.1) and among only blue-collar workers was 3.3 (95% CI, 1.5, 5.0).

### Sensitivity Analyses

The OR for ever-exposure to asbestos in men remained stable when omitting one study at a time; the highest OR, 1.27 (95% CI, 1.19, 1.34), was observed when LUCAS was omitted, and the lowest OR, 1.21 (95% CI, 1.14, 1.29), when AUT-Munich was omitted (data not shown).

When excluding one industry or occupation at a time, the lung cancer risk in the highest quartile of cumulative exposure in men (>2.8 ff/ml-years; OR, 1.38; 95% CI, 1.27, 1.50) remained elevated, as follows: excluding asbestos manufacturing (OR, 1.44; 95% CI, 1.32, 1.57), excluding construction (OR, 1.41; 95% CI, 1.27, 1.57), excluding mining (OR, 1.36; 95% CI, 1.25, 1.49), excluding metal work (OR, 1.43; 95% CI, 1.31, 1.56), excluding transportation (OR, 1.42; 95% CI, 1.30, 1.56), or excluding vehicle mechanic (OR, 1.46; 95% CI, 1.34, 1.60) (data not shown).

Further sensitivity analyses in men (Table 3) on cumulative asbestos exposure showed that stratifying the analyses by studies with population- and hospital-based controls made a difference; ORs in studies with hospital-based controls were generally lower and more imprecise. Restricting the study population to blue-collar workers resulted in a systematic attenuation of the OR by about 10%–15%, although the significant exposure–response trend persisted. Restricting the study population to workers who started working after 1960 also lowered the ORs, whereas excluding “laborers not elsewhere classified” (ISCO 9–99.10) did not markedly influence the overall results.

**TABLE 3.**
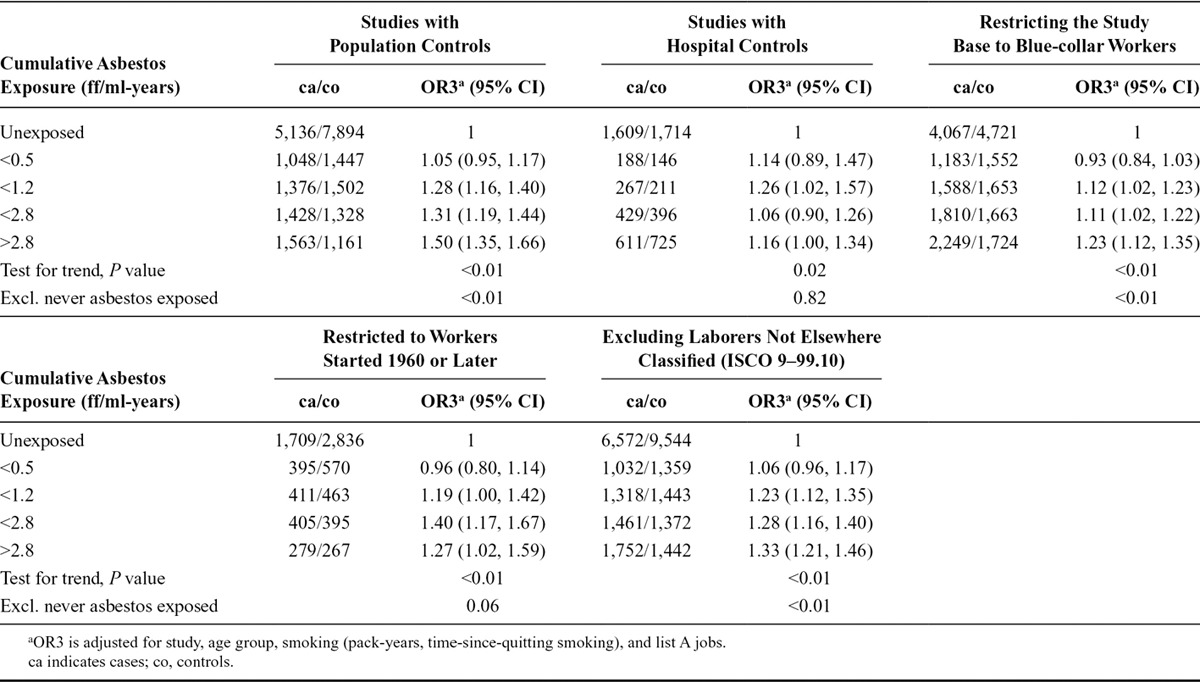
Lung Cancer ORs and 95% CIs in Relation to Cumulative Asbestos Exposure in Restricted Strata (Sensitivity Analyses) of Men in the SYNERGY Study, 1985–2010

### Lung Cancer Risk Associated with Cumulative Asbestos Exposure, Stratified by Major Histological Subtype and Smoking Status

Table 4 shows ORs associated with cumulative asbestos exposure by major lung cancer subtype and by smoking status. Occupational asbestos exposure in men was associated with an increased lung cancer risk among never-smokers, former smokers, and current smokers. Never-smokers with exposure above the median (>1.2 ff/ml-years) had slightly higher ORs than former or current smokers, particularly for small-cell carcinoma (OR, 2.73; 95% CI, 1.39, 5.35). ORs were higher for squamous and small-cell carcinoma than for lung adenocarcinoma (*P* = 0.11 for the likelihood ratio test of homogeneity from the multinomial logistic regression model when these three subtypes were included). In women, stratifying by smoking status and lung cancer subtype revealed associations in subgroups. Among current smokers, we observed associations of asbestos exposure with all lung cancer subtypes, with all ORs increased approximately two-fold. In former smokers, none of the associations was increased; and among never-smokers, our results showed no association for lung adenocarcinoma or squamous-cell lung cancer but a relatively strong association for small-cell lung cancer even at low levels of asbestos exposure (<1.2 ff/ml-years: OR, 3.51; 95% CI, 1.29, 9.55).

**TABLE 4.**
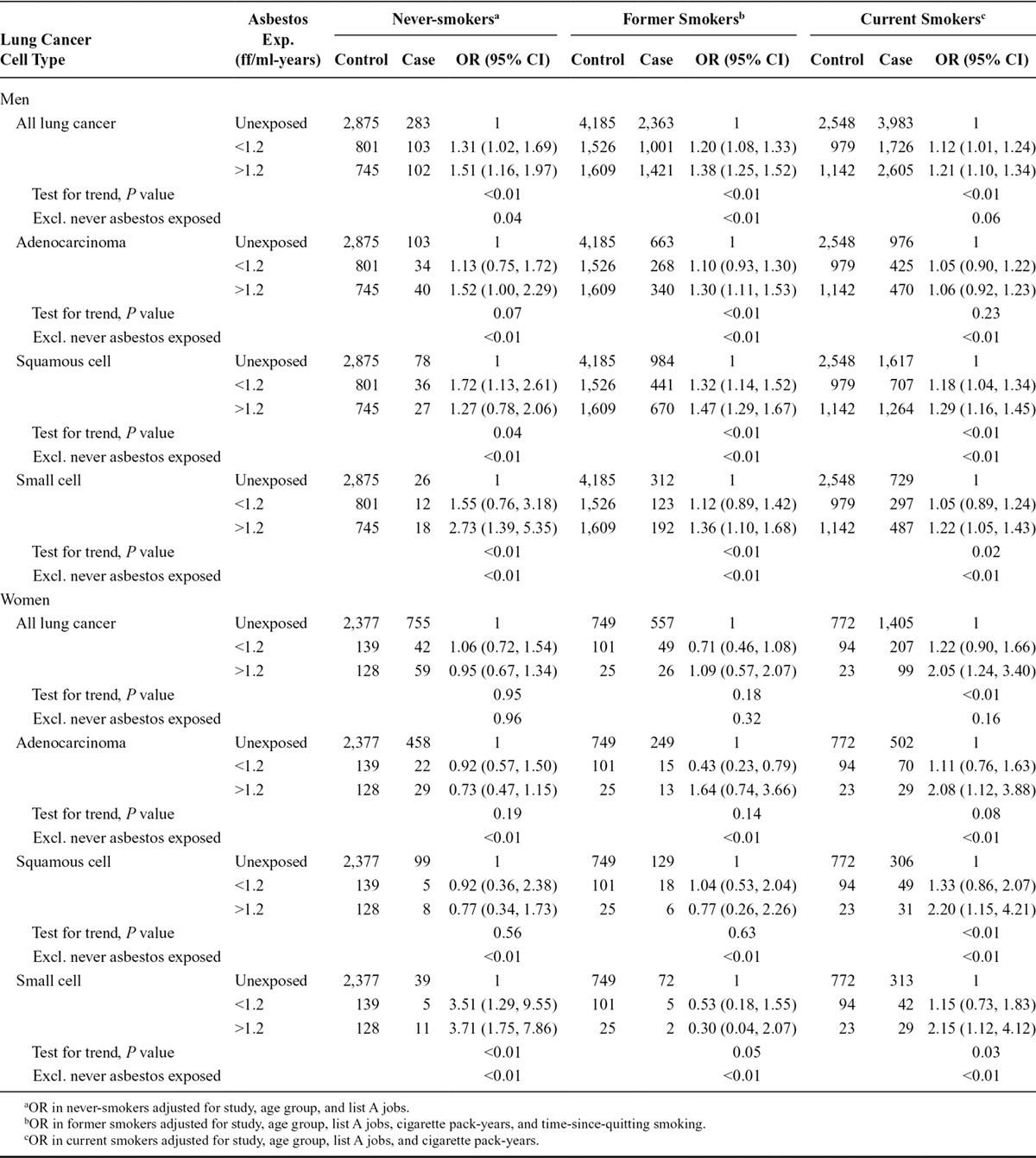
Lung Cancer ORs and 95% CIs in Relation to Cumulative Asbestos Exposure Stratified by Lung Cancer Cell Type and Smoking Status Among Men and Women in the SYNERGY Study, 1985–2010

### Joint Effects of Asbestos and Smoking

Table 5 shows the joint effects of asbestos exposure and smoking, overall and by lung cancer subtype. In men, the joint effect of smoking and asbestos was more than additive for all lung cancer subtypes, with a higher excess risk due to interaction for squamous- and small-cell lung carcinoma than for lung adenocarcinoma, whereas there was no deviation from a multiplicative scale (*P* = 0.10–0.90). Patterns were similar in women, but the RERIs were not significantly different from 0, except for squamous-cell lung carcinoma. The strong association between asbestos exposure and small-cell lung carcinoma in never-smokers resulted in a submultiplicative interaction with smoking, in women (*P* = 0.01) but not in men (*P* = 0.10). A complementary table including ORs for models with and without interaction between occupational asbestos exposure and smoking is shown in eTable 6 (http://links.lww.com/EDE/B144).

**TABLE 5.**
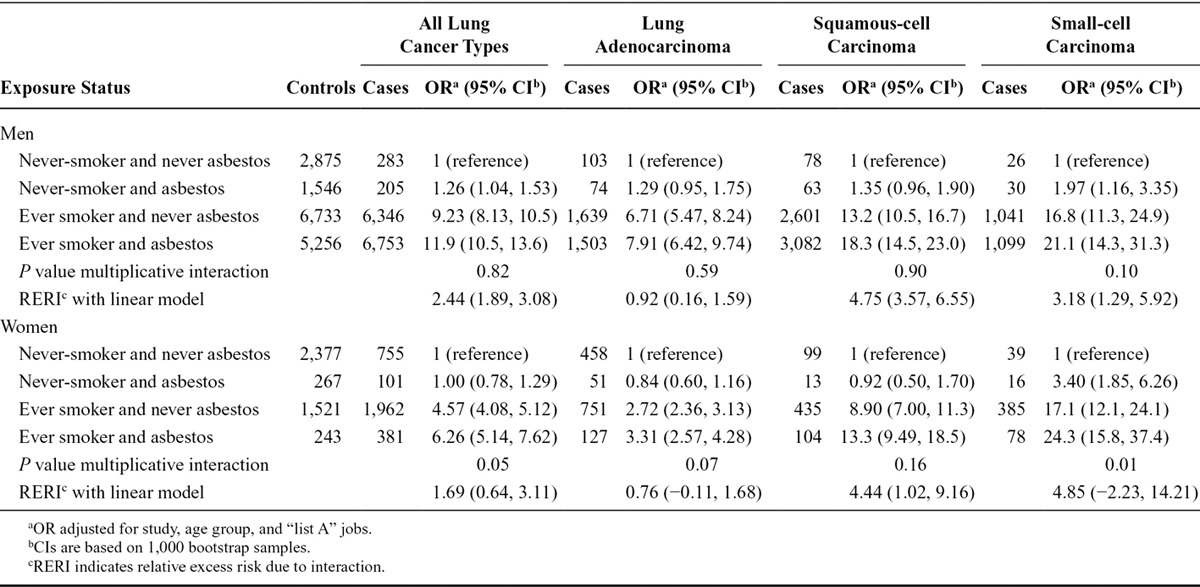
Lung Cancer ORs and 95% CIs, P Value for Multiplicative Interaction and RERI and 95% CI in Relation to Occupational Asbestos Exposure and Smoking Among Men and Women Overall and by Major Lung Cancer Cell Types, in the SYNERGY Study, 1985–2010

## DISCUSSION

We investigated the quantitative association between occupational asbestos exposure and lung cancer risk in the SYNERGY project by sex, smoking status, and lung cancer subtype. Increasing duration and increasing cumulative asbestos exposure were associated with an increasing lung cancer risk in men. Moreover, the increased lung cancer risk in men was observed in never-smokers, former smokers, and current smokers, and for all three major lung cancer subtypes. Sensitivity analyses revealed that results were not driven by exposure in any particular industry or study. Women were exposed to lower levels of asbestos than men (median, 0.57 vs. 1.21 ff/ml-years), which may explain the weaker association with lung cancer among women. The interaction between asbestos exposure and smoking was more than additive for all major lung cancer subtypes among men and for squamous-cell lung carcinoma among women; moreover, the interaction between asbestos and smoking among men did not deviate from multiplicativity. The results from our pooled analysis of case–control studies are in broad agreement with those obtained by Wraith and Mengersen^12^ in a meta-analysis of industry-based cohort and case–control studies, although our study adds results on the interaction between smoking and asbestos by lung cancer subtype.

Lagged exposure estimates generated very similar results (not shown) to those of unlagged estimates; a possible explanation is that the relative exposure distribution remained the same because most exposed subjects were exposed to no or low exposure levels in recent decades, particularly after the implementation of asbestos bans in the different countries. Also, many workers had been retired for many years when they were diagnosed with lung cancer, and therefore their exposure did not change much, even when lagged.

The quality of the exposure assessment has a strong influence on the estimation of the exposure–response association.^23^ So far, quantitative estimates based on measurements have been obtained mainly from industrial cohort studies.^24^ We assessed asbestos exposure applying SYN-JEM for general population studies, a newly created job-exposure matrix based on quantitative workplace measurements from Europe and Canada.

Strengths of this study include the large study population, a large proportion of face-to-face interviews conducted by trained interviewers, a large proportion of control subjects recruited from the general population, a comprehensive adjustment for smoking, and an innovative and objective method, supported by actual measurements, for assessing asbestos exposure quantitatively in general population studies.

Limitations of the study are that asbestos fiber type and dimensions could not be taken into account, because almost all measurements (>95%) were determined by phase-contrast microscopy, which does not allow the fiber type to be distinguished or to identify fibers with width <0.25 μm. For lung cancer, scientific uncertainty remains on how much risks differ in magnitude by fiber type, but recent evidence suggests it is less than previously assumed.^24,25^

Misclassification of exposure is assumed to have occurred, but it can also be assumed to be nondifferential provided reporting of job titles did not differ systematically in cases and controls. Differential reporting of job titles is not likely; therefore, the use of a job-exposure matrix for exposure assessment is unlikely to have created spurious associations.

Further limitations include that measurements were not done for individual study subjects, resulting in assignments of average exposure levels to job titles and not to individuals, and leading to assignment of the same exposure level to all workers sharing the same job code in a particular year, irrespective of exposure variability between workers in the same job. This results in a Berkson-type error, which usually does not bias the point estimate but increases the variance and therefore leads to reduced precision.^26^

Some jobs or unspecific job codes may substantially influence the prevalence of exposure if used extensively in a study. For example, many jobs were coded as “laborers not elsewhere classified” (ISCO 9–99.10); in SYN-JEM, this job was assigned low exposure to asbestos. The use of the ISCO 9–99.10 job code does not necessarily reflect poor quality of interviews or coding, as it could also signify a high prevalence of low specialization of laborers and/or low technology in certain industries. Indeed, the asbestos prevalence decreased by >10% in some studies when excluding “laborers not elsewhere classified.” Nevertheless, excluding “laborers not elsewhere classified” from the risk analyses did not change the overall results, possibly because 9–99.10 jobs represented only 1.8% of the total working time.

Another limitation is that half of the 36,000 personal measurements collected were available for the production of asbestos cement and asbestos textiles, job titles that were rare in our study population or too specific to be captured by the ISCO code.^15^ In the SYNERGY study population, only 46 subjects (32 cases, 14 controls) had ever worked as asbestos cement product makers. The number of available data points for the remaining, more prevalent jobs was more limited.

Exposure assessment according to SYN-JEM resulted in high prevalence of ever occupational asbestos exposure compared with the original studies.^27–30^ However, the prevalence of asbestos exposure decreased substantially, from 41.3% to 6.4% among male controls and from 11.3% to 0.3% among female controls, when only the DOM-JEM high-exposure jobs were considered, which confirms that the vast majority of exposed workers were employed in jobs with low average exposure levels.

About 60% of all blue-collar workers were rated as ever-exposed to asbestos. This implies that they may have been exposed to many other agents at their workplaces. Although we controlled for occupations with known exposure to pulmonary carcinogens, there may still remain some uncertainty about residual confounding by other occupational hazards. Notably, the association between asbestos exposure and lung cancer risk was weaker when restricting to blue-collar workers. This may be due to a reduction in exposure contrast. Alternatively, the background lung cancer risk may be higher in blue-collar workers than in white-collar workers due to other agents in the workplace and various socioeconomic factors.^31–33^ Also, selection bias has to be taken into account due to a lower participation rate of blue-collar workers among population controls.^34^ The hospital-based studies showed lower ORs for asbestos exposure than the population-based studies, which may be explained by a combination of factors, including choice of control diseases, geographical location (possibly reflecting different exposure patterns), study size, or other factors.

Levels of occupational asbestos exposure in this pooled analysis of general population studies were lower (range, 0.0023–64.6 ff/ml-years in male controls) than in the 18 industrial cohort studies (range, 0.11–4,710 ff/ml-years) included in a recent review of exposure–response relationships.^24^ A probable reason for the rather low levels of occupational asbestos exposure seen in SYNERGY is that major “asbestos occupations” such as asbestos cement product makers and asbestos textile workers are rare in a general population study setting. Instead, exposures in SYNERGY represent a wider exposure range, with very few workers exposed to high levels and most being downstream users or indirectly exposed workers occasionally exposed or exposed to lower concentrations of fibers only.

Our large dataset may be particularly informative to explore the shape of the exposure–response function in the low-dose range. An additional advantage was stratification or detailed adjustment for smoking. In our analysis of pooled general population studies, the exposure–response slope estimated as excess risk per 100 fiber-years (*K*_*L*_ * 100) was 6.1 (95% CI, 4.1, 8.1) in men overall and 3.3 (95% CI, 1.5, 5.0) among male blue-collar workers. The *K*_*L*_ * 100 slope was flat in a recent meta-analysis of 18 occupational cohort studies (*K*_*L*_ * 100, 0.13; 95% CI, 0.04, 0.22), while it was rather steep (*K*_*L*_ * 100, 15.5; 95% CI, 1.13, 29.87) in LUCAS, the general population study from Stockholm, which also is part of SYNERGY.^24,35^ The SYNERGY *K*_*L*_ * 100 estimate in blue-collar workers (3.3; 95% CI, 1.5, 5.0) is still considerably higher than the estimate from the industrial cohorts in Lenters’ paper, which were considered to have good-quality exposure assessment (e.g., seven cohort studies with >30% coverage of exposure data; *K*_*L*_ * 100, 0.27; 95% CI, 0.08, 0.46). A possible reason for the steeper slope we observed in general population studies is that we could assess the full occupational history, resulting in a more distinct exposure contrast, and that we could identify a substantial proportion of truly nonexposed. However, there is little evidence in the literature regarding the shape of the exposure–response curve at low levels of exposure.^25,36^ Our dataset is less informative regarding the high-dose range; only 90 of 29,997 male participants were assessed as exposed to ≥15 fiber-years. The exposure–response results presented here are based on exposure–response modeling agreed upon a priori. This model assumes ln(OR) is proportional to ln(exposure). Other exposure–response models will result in different risk estimates.

We observed a stronger association between asbestos exposure and small-cell lung carcinoma among never-smokers in both men and women. This is noteworthy because small- and squamous-cell lung carcinomas occur almost exclusively in cigarette smokers; in SYNERGY, only 4% of small- and squamous-cell cases were never-smokers, whereas 14% of lung adenocarcinoma cases were never-smokers. However, we cannot rule out biased recall of smoking habits.^37^

Some misclassification of the histological subtypes of lung cancer is likely; one of the studies (HdA) included in SYNERGY assessed diagnostic agreement between pathologists and found a kappa of 0.54 (95% CI, 0.49, 0.58).^14^ Small-cell lung cancer was best classified, followed by squamous-cell lung cancer and lung adenocarcinoma. Most misclassification was between squamous-cell lung cancer and lung adenocarcinoma. Thus, our results for the major lung cancer subtypes should be interpreted with caution.

Our results show an excess risk of lung cancer and its subtypes at relatively low levels of cumulative exposure (>0.5 ff/ml-years), which persisted at least up to 40 years after last exposure. Furthermore, the slope of the exposure–response relationship seemed steeper in this exposure range than at higher (and previously studied) levels. Together, this implies that the future burden of disease due to asbestos exposure may be underestimated.

## ACKNOWLEDGMENTS

The authors wish to thank Guillermo Fernandez-Tardon, hygienist at the University Institute of Oncology of Asturias - Cajastur Social Programme (IUOPA), in Oviedo, Asturias.

## Supplementary Material
