# Supplementary material for: Exposure–Response Analyses of Asbestos and Lung Cancer Subtypes in a Pooled Analysis of Case–Control Studies
Source: Epidemiology. 2017 Feb 1;28(2):288–99. doi: 10.1097/EDE.0000000000000604 (PMC5287435; doi:10.1097/EDE.0000000000000604)
Supplement: Supplementary file 1 [file ede-28-288-s001.docx]

**eTable 1.** Description of the studies included in these analyses in the SYNERGY project

| **Study** | **Country** | **Data collection** | **Cases** | | **Controls** | |  | **Source of controls P=Population H=Hospital** | **Interviewee S=subject** |
| --- | --- | --- | --- | --- | --- | --- | --- | --- | --- |
|  |  |  | **N** | **Response rate (%)** | **N** | **Response rate (%)** | **Asbestos exposure** |  | **NOK=Next- of-kin** |
| AUT-Munich | Germany | 1990–1995 | 3180 | 77 | 3249 | 41 | 1946-1995 | P | S |
| HdA | Germany | 1988–1993 | 1004 | 69 | 1004 | 68 | 1946-1993 | P | S |
| EAGLE | Italy | 2002–2005 | 1943 | 87 | 2116 | 72 | 1936-2005 | P | S |
| TURIN/VENETO | Italy | 1990–1994 | 1132 | 79 | 1553 | 80 | 1922-1994 | P | S |
| ROME | Italy | 1993–1996 | 347 | 74 | 365 | 63 | 1929-1996 | H | S |
| LUCA | France | 1989–1992 | 309 | 98 | 302 | 98 | 1946-1992 | H | S |
| PARIS | France | 1988–1992 | 173 | 95 | 234 | 95 | 1946-1992 | H | S |
| ICARE | France | 2001–2007 | 2926 | 87 | 3555 | 81 | 1946-2007 | P | S & NOK |
| CAPUA | Spain | 2000–2010 | 875 | 91 | 838 | 96 | 1946-2009 | H | S |
| MORGEN^a^ | Netherlands | 1993–1997 | 71 | N/A | 202 | N/A | 1946-1995 | P | S |
| INCO | Czech Republic | 1999–2002 | 304 | 94 | 453 | 80 | 1946-2002 | H | S |
| INCO | Hungary | 1998–2001 | 402 | 90 | 315 | 100 | 1946-1999 | H | S |
| INCO | Poland | 1998–2002 | 800 | 88 | 841 | 88 | 1946-2001 | P & H | S |
| INCO | Slovakia | 1998–2002 | 346 | 90 | 285 | 84 | 1946-2002 | H | S |
| INCO | Romania | 1998–2002 | 181 | 90 | 228 | 99 | 1946-2002 | H | S |
| INCO | Russia | 1998–2001 | 600 | 96 | 580 | 90 | 1936-2000 | H | S |
| INCO/LLP | United Kingdom | 1998–2005 | 442 | 78 | 918 | 84 | 1933-2004 | P | S |
| LUCAS | Sweden | 1985–1990 | 1042 | 87 | 2356 | 85 | 1946-1990 | P | S & NOK |
| MONTREAL | Canada | 1996–2002 | 1203 | 85 | 1509 | 69 | 1935-2002 | P | S & NOK |
| TORONTO | Canada | 1997–2002 | 425 | 62 | 910 | 71 | 1933-2002 | P & H | S |
| Overall | 14 countries | 1985–2010 | 17705 | 83% | 21813 | 70% | 1922-2009 | H=21% | NOK=7.3% |

^a^ Nested case-control study: 45% of invited participants to the original cohort completed the baseline questionnaire

**eTable 2.** Prevalence of occupational asbestos exposure among control subjects in men by study before and after excluding the category “laborers not elsewhere classified (n.e.c.)” (ISCO 9-99.XX), and ever high asbestos exposure

| **Study** | **All male control subjects** | | **Excl. labourer n.e.c. (ISCO 9-99.xx)** | | **All male control subjects** | |
| --- | --- | --- | --- | --- | --- | --- |
|  | Asbestos exposure | | Asbestos exposure | | High asbestos exposure  (DOM-JEM=2) | |
|  | No. | % | No. | % of all | No. | % of all |
| AUT-Munich | 1285 | 47.6 | 1100 | 40.7 | 205 | 7.6 |
| CAPUA | 197 | 43.3 | 151 | 33.2 | 19 | 4.2 |
| EAGLE | 500 | 31.6 | 477 | 30.1 | 71 | 4.5 |
| HdA | 434 | 51.9 | 310 | 37.1 | 96 | 11.5 |
| ICARE | 1060 | 39.2 | 940 | 34.8 | 182 | 6.7 |
| INCO_CZ | 141 | 48.1 | 135 | 46.1 | 19 | 6.5 |
| INCO_Hungary | 127 | 51.6 | 94 | 38.2 | 22 | 8.9 |
| INCO_Poland | 220 | 38.4 | 191 | 33.3 | 18 | 3.1 |
| INCO_Romania | 46 | 30.5 | 43 | 28.5 | 4 | 2.6 |
| INCO_Russia | 288 | 57.3 | 237 | 47.1 | 45 | 8.9 |
| INCO_Slovakia | 94 | 39.8 | 76 | 32.2 | 25 | 10.6 |
| INCO/LLP_UK | 359 | 62.8 | 263 | 46.0 | 72 | 12.6 |
| LUCA | 110 | 39.0 | 90 | 31.9 | 20 | 7.1 |
| LUCAS | 614 | 26.6 | 569 | 24.7 | 89 | 3.9 |
| MONTREAL | 412 | 46.1 | 301 | 33.7 | 45 | 5.0 |
| MORGEN | 12 | 21.1 | 12 | 21.1 | 2 | 3.5 |
| PARIS | 102 | 47.2 | 77 | 35.6 | 18 | 8.3 |
| ROME | 118 | 45.9 | 74 | 28.8 | 12 | 4.7 |
| TURIN/VENETO | 616 | 49.9 | 414 | 33.5 | 76 | 6.2 |
| TORONTO | 67 | 19.3 | 58 | 16.7 | 6 | 1.7 |
| **TOTAL** | **6802** | **41.3** | **5612** | **34.1** | **1046** | **6.4** |

**eTable 3.** Prevalence of occupational asbestos exposure among control subjects in women by study before and after excluding the category “laborers not elsewhere classified (n.e.c.)”(ISCO 9-99.XX), and ever high asbestos exposure

| **Study** | **All female control subjects** | | **Excl. labourer n.e.c. (ISCO 9-99.xx)** | | **All female control subjects** | |
| --- | --- | --- | --- | --- | --- | --- |
|  | Asbestos exposure | | Asbestos exposure | | High asbestos exposure  (DOM-JEM=2) | |
|  | No. | % | No. | % of all | No. | % of all |
| AUT-Munich | 55 | 10.1 | 41 | 7.5 | 3 | 0.5 |
| CAPUA | 4 | 7.0 | 3 | 5.3 | 0 | 0.0 |
| EAGLE | 47 | 9.7 | 47 | 9.7 | 2 | 0.4 |
| HdA | 23 | 13.9 | 18 | 10.9 | 2 | 1.2 |
| ICARE | 68 | 9.2 | 52 | 7.0 | 1 | 0.1 |
| INCO_CZ | 14 | 8.8 | 13 | 8.2 | 0 | 0.0 |
| INCO_Hungary | 11 | 18.6 | 4 | 6.8 | 0 | 0.0 |
| INCO_Poland | 25 | 9.5 | 23 | 8.8 | 0 | 0.0 |
| INCO_Romania | 9 | 12.2 | 8 | 10.8 | 0 | 0.0 |
| INCO_Russia | 26 | 33.8 | 11 | 14.3 | 0 | 0.0 |
| INCO_Slovakia | 5 | 10.2 | 4 | 8.2 | 2 | 4.1 |
| INCO/LLP_UK | 119 | 34.6 | 116 | 33.7 | 1 | 0.3 |
| MONTREAL | 47 | 7.7 | 46 | 7.5 | 2 | 0.3 |
| MORGEN | 0 | 0.0 | 0 | 0.0 | 0 | 0.0 |
| PARIS | 1 | 9.1 | 1 | 9.1 | 1 | 9.1 |
| ROME | 4 | 6.3 | 3 | 4.7 | 0 | 0.0 |
| TURIN/VENETO | 30 | 11.8 | 24 | 9.4 | 0 | 0.0 |
| TORONTO | 22 | 4.4 | 18 | 3.6 | 0 | 0.0 |
| **TOTAL** | **510** | **11.3** | **432** | **9.6** | **14** | **0.3** |


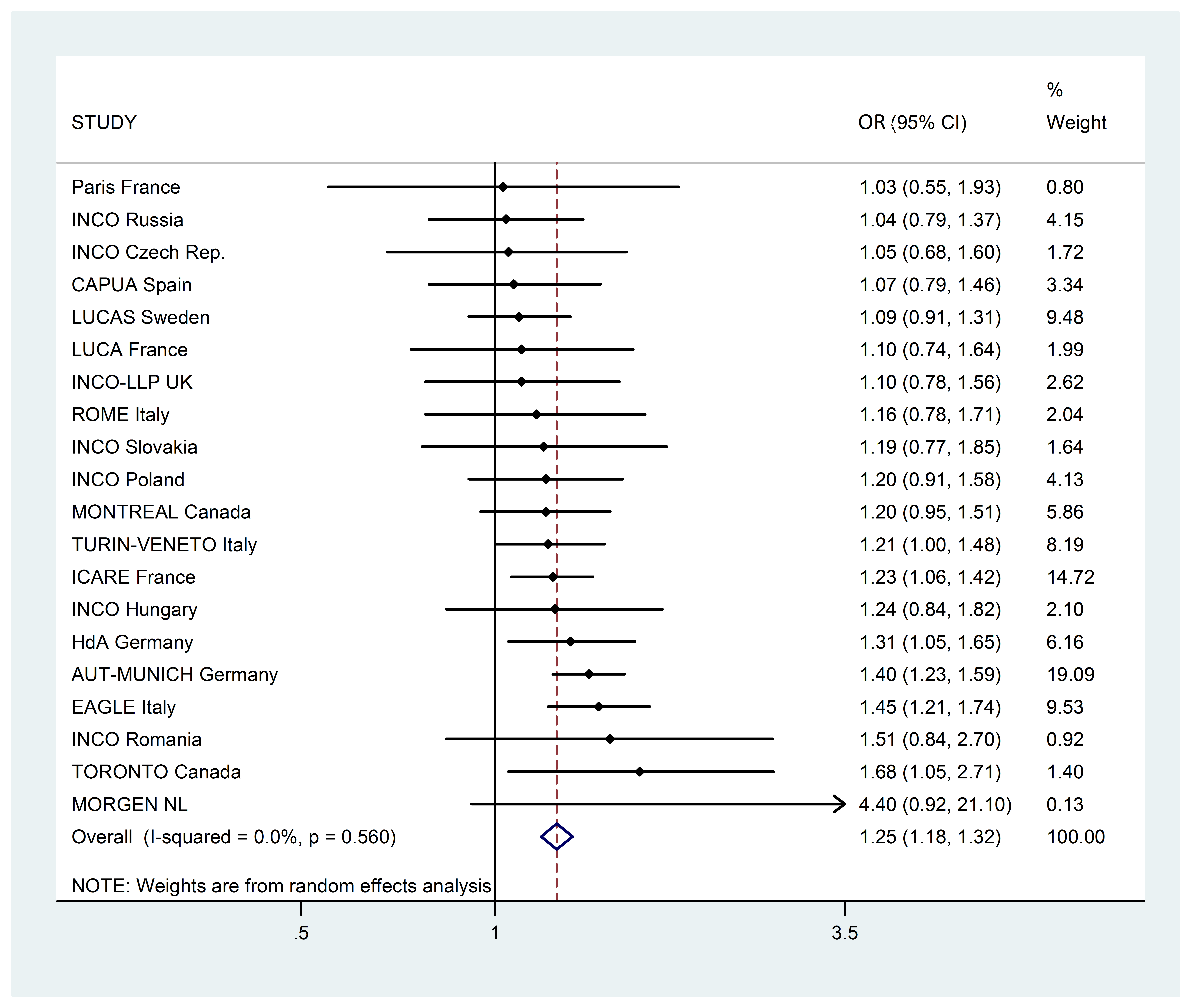


**eFigure 4.** Study-specific odds ratios (OR3) for ever-exposure to asbestos compared with never-exposed in men adjusted for age group, cigarette pack-years, time-since-quitting smoking, and ever-employment in a “List A” job


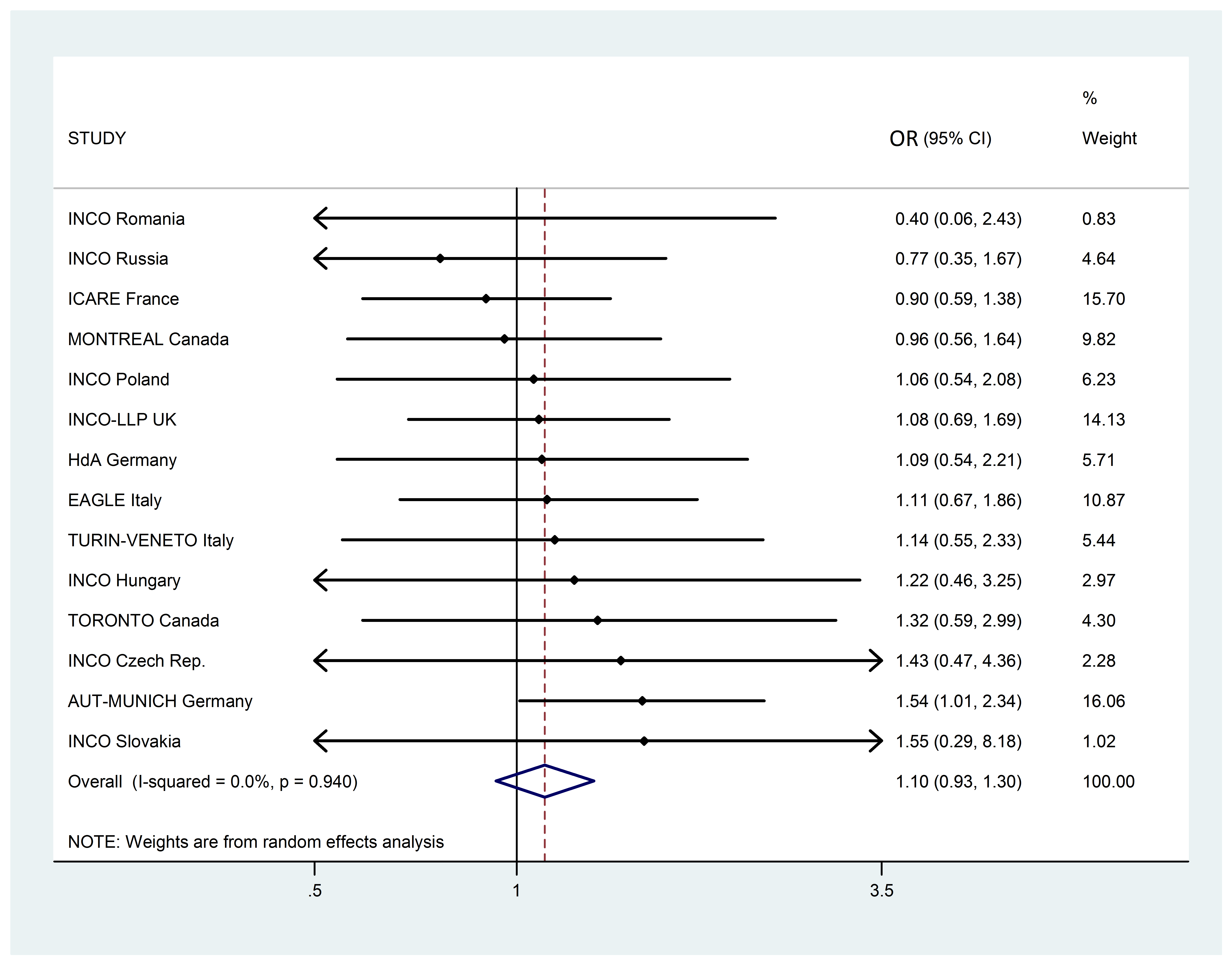


**eFigure 5.** Study-specific odds ratios (OR3) for ever exposure to asbestos compared with never-exposed in women adjusted for age group, cigarette pack-years, time-since-quitting smoking, and ever-employment in a “List A” job

| **eTable 6. Lung cancer odds ratios for models with and without interaction between occupational asbestos exposure and smoking among men and women overall and by major lung cancer cell types, in the SYNERGY study, 1985–2010** | | | | | | | |
| --- | --- | --- | --- | --- | --- | --- | --- |
| **Lung cancer cell type** | **Exposure categories asbestos** | **Men** | | | **Women** | | |
|  |  | OR (SE) | with Interaction | P value ^a^ | OR (SE) | with Interaction | P-value ^a^ |
| All lung cancers | Exposed | 1.27 (1.03) | 1.23 (1.09) | 0.74 | 1.13 (1.07) | 1.06 (1.14) | 0.55 |
|  | Ever Smoker | 8.08 (1.05) | 8.00 (1.06) |  | 4.31 (1.05) | 4.26 (1.06) |  |
|  | Exposed * Ever Smoker |  | 1.03 (1.09) |  |  | 1.09 (1.16) |  |
| Squamous cell lung cancer | Exposed | 1.36 (1.03) | 1.30 (1.17) | 0.72 | 1.20 (1.13) | 0.97 (1.35) | 0.44 |
|  | Ever Smoker | 11.02 (1.08) | 10.70 (1.11) |  | 8.08 (1.12) | 7.85 (1.13) |  |
|  | Exposed * Ever Smoker |  | 1.06 (1.17) |  |  | 1.28 (1.39) |  |
| Small cell lung cancer | Exposed | 1.22 (1.05) | 1.70 (1.28) | 0.18 | 1.32 (1.15) | 3.22 (1.36) | 0.002 |
|  | Ever Smoker | 11.47 (1.13) | 13.33 (1.20) |  | 13.60 (1.16) | 16.78 (1.19) |  |
|  | Exposed * Ever Smoker |  | 0.72 (1.28) |  |  | 0.35 (1.40) |  |
| Adenocarcinoma | Exposed | 1.17 (1.04) | 1.30 (1.16) | 0.48 | 1.02 (1.11) | 0.91 (1.17) | 0.37 |
|  | Ever Smoker | 5.87 (1.08) | 6.11 (1.11) |  | 2.61 (1.07) | 2.56 (1.07) |  |
|  | Exposed * Ever Smoker |  | 0.90 (1.16) |  |  | 1.20 (1.22) |  |
|  |  |  |  |  |  |  |  |
| 'Never Smokers' and 'Not asbestos exposed' are the reference categories  OR are adjusted for study, age-group, ever-employment in List A job. | | | | | | |  |
| ^a^ P-value on Interaction variable | |  |  |  |  |  |  |
